# Gene Editing and Crop Improvement Using CRISPR-Cas9 System

**DOI:** 10.3389/fpls.2017.01932

**Published:** 2017-11-08

**Authors:** Leena Arora, Alka Narula

**Affiliations:** Department of Biotechnology, School of Chemical and Life Sciences, Jamia Hamdard University, New Delhi, India

**Keywords:** CRISPR/Cas system, genome editing, nutrition improvement, disease resistance, metabolic engineering, gene expression regulation, CRISPR ribonucleoproteins

## Abstract

Advancements in Genome editing technologies have revolutionized the fields of functional genomics and crop improvement. CRISPR/Cas9 (clustered regularly interspaced short palindromic repeat)-Cas9 is a multipurpose technology for genetic engineering that relies on the complementarity of the guideRNA (gRNA) to a specific sequence and the Cas9 endonuclease activity. It has broadened the agricultural research area, bringing in new opportunities to develop novel plant varieties with deletion of detrimental traits or addition of significant characters. This RNA guided genome editing technology is turning out to be a groundbreaking innovation in distinct branches of plant biology. CRISPR technology is constantly advancing including options for various genetic manipulations like generating knockouts; making precise modifications, multiplex genome engineering, and activation and repression of target genes. The review highlights the progression throughout the CRISPR legacy. We have studied the rapid evolution of CRISPR/Cas9 tools with myriad functionalities, capabilities, and specialized applications. Among varied diligences, plant nutritional improvement, enhancement of plant disease resistance and production of drought tolerant plants are reviewed. The review also includes some information on traditional delivery methods of Cas9-gRNA complexes into plant cells and incorporates the advent of CRISPR ribonucleoproteins (RNPs) that came up as a solution to various limitations that prevailed with plasmid-based CRISPR system.

## Introduction

**FIGURE 1:**
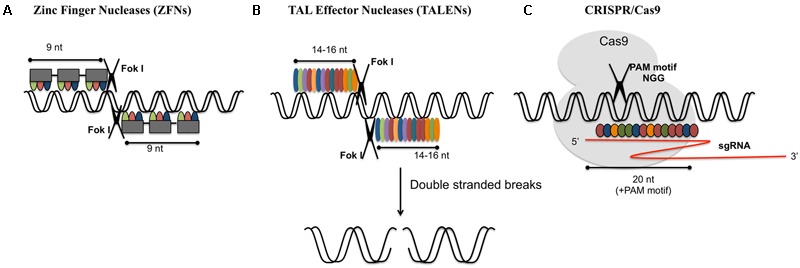
Various genome-editing tools. **(A)** Zinc-finger nucleases (ZFNs) act as dimer. Each monomer consists of a DNA binding domain and a nuclease domain. Each DNA binding domain consists of an array of 3–6 zinc finger repeats which recognizes 9–18 nucleotides. Nuclease domain consists of type II restriction endonuclease Fok1. **(B)** Transcription activator-like nucleases (TALENs): these are dimeric enzymes similar to ZFNs. Each subunit consists of DNA binding domain (highly conserved 33–34 amino acid sequence specific for each nucleotide) and Fok1 nuclease domain. **(C)** CRISPR/Cas9: Cas9 endonuclease is guided by sgRNA (single guide RNA: crRNA and tracrRNA) for target specific cleavage. 20 nucleotide recognition site is present upstream of protospacer adjacent motif (PAM).

Genetic diversity is a key source for trait improvement in plants. Creating variations in the gene pool is the foremost requirement for developing novel plant varieties. Once the desired alterations are achieved, transgenes can be crossed out from the improved variety. Crop improvement has been done for years via traditional plant breeding techniques or through various physical, chemical (e.g., gamma radiation, ethyl methanesulfonate) and biological methods (e.g., T-DNA, transposon insertion) leading to point mutations, deletions, rearrangements, and gene duplications. The advent of site-specific nucleases (SSNs) highlighted the importance of site directed mutagenesis over random mutagenesis (Osakabe et al., 2010; Sikora et al., 2011). Random mutagenesis has also its own list of shortcomings too. It produces multiple undesirable rearrangements and mutations, which are expensive and very complex to screen. Gene editing uses engineered SSNs to delete, insert or replace a DNA sequence. Development of the engineered endonucleases/mega-nucleases, zinc finger nucleases (ZFNs), transcription activator-like effector nucleases (TALENs) and type II clustered regularly interspaced short palindromic repeat (CRISPR)/CRISPR-associated protein 9 (Cas9) paved the way for single nucleotide excision mechanism for crop improvement (Pabo et al., 2001; Boch et al., 2009; Moscou and Bogdanove, 2009) (**Figure 1**). These genome-editing technologies use programmable nucleases to increase the specificity of the target locus.

Genome editing modifies a specific genome in precise and predictable manner. There could be varieties of genes, which could be altered in different cell types and organisms with the aid of nucleases that offer targeted alterations. ZFNs is one of the oldest gene editing technologies, developed in the 1990s and owned by Sangamo BioSciences. ZFNs are premeditated restriction enzymes having sequence specific DNA binding zinc finger motifs and non-specific cleavage domain of Fok1 endonuclease. An array of 4–6 binding modules combines to form a single zinc finger unit. Each module recognizes a codon (Pabo et al., 2001). A pair of ZFNs together identifies a unique 18–24 bp DNA sequence and double stranded breaks (DSBs) are made by Fok1 dimer. FokI nucleases are naturally occurring type IIS restriction enzymes that introduce single stranded breaks in a double helical DNA. Hence FokI functions as a dimer, with each catalytic monomer (nickase) cleaving a single DNA strand to create a staggered DSB with overhangs (Pabo et al., 2001). ZFNs have been successfully employed in genome modification of various plants including tobacco, maize, soybean, etc. (Curtin et al., 2011; Ainley et al., 2013; Baltes et al., 2014). It was taken back due to some drawbacks such as time-consuming and expensive construction of target enzymes, low specificity and high off-target mutations that eventually made way for the new technology. TALENs turned out to be a substitute to ZFNs and were identified as restriction enzymes that could be manipulated for cutting specific DNA sequences. Traditionally, TALENs were considered as long segments of transcription activator-like effector (TALE) sequences that occurred naturally and joined the Fokl domain with carboxylic-terminal end of manipulated TALE repeat arrays (Christian et al., 2010). TALENs contain a customizable DNA-binding domain which is fused with non-specific Fokl nuclease domain (Christian et al., 2010). TALENs compared to ZFNs, involve the interaction of individual nucleotide repeats of the target site and amino acid sequences of TAL effector proteins. They can generate overhangs by employing Fokl nuclease domain to persuade site-specific DNA cleavage. It has been widely used to generate non-homologous mutations with higher efficiencies in diverse organisms (Joung and Sander, 2012).

**FIGURE 2:**
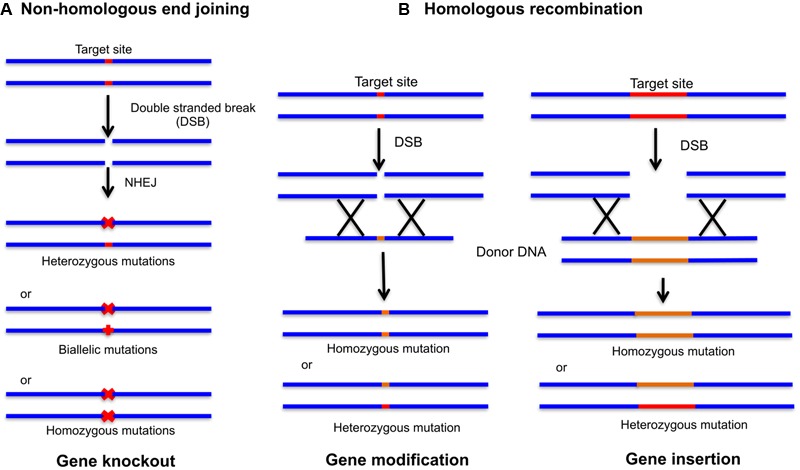
Genome editing with site-specific nucleases (SSNs). The double stranded breaks (DSBs) introduced by CRISPR/Cas9 complex can be repaired by non-homologous end joining (NHEJ) and homologous recombination (HR). **(A)** NHEJ repair can produce heterozygous mutations, biallelic mutations (two different mutations at each chromosome) and homozygous mutations (two independent identical mutations) leading to gene insertion or gene deletion. **(B)** In the presence of donor DNA digested with the same endonuclease leaving behind similar overhangs, HR can be achieved leading to gene modification and insertion.

The emergence of CRISPR technology supersedes ZFNs and TALENs and used widely as a novel approach from “methods of the year” in 2011 to “breakthrough of the year” in 2015 for their captivated genome editing. This prokaryotic system is promptly accepted for genome editing in eukaryotic host cells (Jinek et al., 2012; Nakayama et al., 2013). CRISPR has an added advantage of gene knockout over RNAi, which is a well-known technique for gene knockdown. CRISPR targets the endogenous genes that are impossible to specifically target using RNAi technology with more precision and simplicity. RNAi gene regulation is governed by the endogenous microRNAs (miRNAs). Any displacement of these miRNAs from the exogenous miRNAs can lead to hypomorphic mutations and off-target phenotypes (Khan et al., 2009). CRISPR/Cas9 targets specific genomic loci with the help of ∼100 nucleotide (nt) guide RNA (gRNA) sequence. sgRNA binds to the protospacer adjacent motif (PAM) on targeted DNA via Watson and Crick base pairing through 17–20 nt at the gRNA 5′-end and guide Cas9 for specific cleavage (Tsai et al., 2015). Cas9 stimulates the DNA repair mechanism by introducing DSBs in the target DNA. Repair mechanism involves error prone non-homologous end joining (NHEJ) or homologous recombination (HR) to produce genomic alterations, gene knockouts and gene insertions (**Figure 2**). NHEJ by far is the most common DSB repair mechanism in somatic plant cells (Puchta, 2005). Random insertions or deletions by NHEJ in the coding region lead to frame shift mutations, hence creating gene knockouts. CRISPR technology holds potential for loss-of-function, gain-of-function, and gene expression analysis. CRISPR has versatile applications in plant biology and is readily applied to produce high quality agriculturally sustainable products (**Table 1**). There are many plants which are in the process of getting altered through CRISPR/Cas9. The CRISPR edited tomatoes will be expected to have enhanced flavor, sugar content and aroma as compared to modern commercial varieties; corn is made resistant to drought with high yield per hectare; wheat is edited against powdery mildew disease, and mushrooms are targeted to reduce the melanin content (Wang et al., 2014; Waltz, 2016; Shi et al., 2017; Tieman et al., 2017).

**Table 1:** List of targeted genes via CRISPR/Cas9 system in different plant species.

Plant species	Target gene; gene function	Cas9 promoter; codon optimization of Cas9	sgRNA promoter	Transformation methods	Multiplex strategy; transformant type	Mutant efficiency; type of mutants	Reference
*Arabidopsis thaliana*	AtPDS3; phytoene desaturase, AtFLS2; flagellin sensitive 2, AtRACK1b, AtRACK1c; receptor for activated C kinase1	CaMV35SPDK; plant	AtU6	PEG Protoplast co-transfection and *Agrobacterium* infiltration	Transient	1.1–5.6%	Li et al., 2013
	CHL1, CHL2; magnesium chelatase subunit1	OsUBQ1; human	OsU3	*Agrobacterium* mediated transformation	Sequential cloning	81.2%; chimeric	Mao et al., 2013
	TRY, CPC, and ETC2; trichome density regulators	P35S; maize	AtU6-26, AtU6-29	Argo-transformation by floral dip	Golden gate/Gibson assembly	82.6%; chimeric	Xing et al., 2014
*Nicotiana benthamiana*	PDS gene; phytoene desaturase	P35S; human	AtU6	Leaf agroinfiltration	Sequential cloning	6.7%; chimeric	Nekrasov et al., 2013
	PDS, PDR6; phytoene desaturase	2 × CaMV3; tobacco	AtU6	PEG protoplast transfection	Transient	16.27–20.3%	Gao and Zhao, 2014
	NtPDR6; ABC transporter	2 × P35S; tobacco	AtU6-26	*Agrobacterium*	Restriction cloning	81.8–87.5%; chimeric	Gao et al., 2015
*Oryza sativa*	OsSWEET; disease susceptibility gene	OsUbi; rice	OsU6.1, OsU6.2	*Agrobacterium* mediated transformation	Sequential cloning	12.5%	Zhou et al., 2014
	OsWaxy; amylose synthase	ZmUbi; rice	OsU3, OsU6a, OsU6b, OsU6c		Golden gate/Gibson assembly	85.4%; biallelic, homozygous, heterozygous	Ma et al., 2015
	*Gn1a, DEP1, GS3*, and *IPA1*; regulators of grain number, panicle architecture	Maize ubiquitin promoter	U6a	Agro-transformation in embryogenic calli	–	42.5% (Gn1a), 67.5% (DEP1), 57.5% (GS3)	Li M. et al., 2016
*Zea mays*	Liguleless1 (LIG1) gene, male fertility genes (Ms26 and Ms45), and acetolactate synthase (ALS) genes (ALS1 and ALS2)	ZmUbi; maize	ZmU6	Biolistic transformation	Co-delivery	77–100%; biallelic, heterozygous	Svitashev et al., 2016
	Argonaute 18, dihydroflavonol 4-reductase	PZmUbi	PU6.1, PU6.2	Protoplast transfection	Sequential cloning	70%	Char et al., 2016
*Triticum aestivum*	TaMLO homologs; repress resistance pathway to powdery mildew	ZmUbi; plant	TaU6	Particle bombardment of immature embryos	–	5.6%	Wang et al., 2014
*Solanum tuberosum*	GBSS; starch synthase gene	CaMV 35S	AtU6, StU6	PEG-mediated protoplast transfection	Restriction cloning	67%	Andersson et al., 2016
*Solanum lycopersicum*	ANT1; Anthocyanin biosynthesis	35S	ANT1; AtU6	*Agrobacterium* mediated transformation	Golden gate	57.1% heterozygous; 13.1% homozygous	Cermak et al., 2015
	SIAGO7; biogenesis of trans-acting short interfering RNAs	P35S; human	AtU6	Agro-transformation of cotyledons	Golden gate	48%; homozygous, biallelic, chimeric	Brooks et al., 2014
*Gossypium hirsutum*	*Cloroplastos alterados 1 (GhCLA1*) and *vacuolar H*+*-pyrophosphatase (GhVP*) genes	2 × 35S	AtU6-26	*Agrobacterium* mediated transformation	Golden gate	47.6–81.8%	Chen et al., 2017
*Sorghum bicolor*	DsRed; red fluorescent protein	CaMV 35S	AtU6	Agro-transformation of immature embryos	Golden gate	28%	Jiang et al., 2013
*Populus tomentosa*	PDS; phytoene desaturase	2 × P35S; plant	AtU3b, AtU3d	*Agrobacterium* mediated transformation	Golden gate	50.9%	Fan et al., 2015
*Glycine max*	ALS1; encode acetolactate synthase involved in amino acid biosynthesis	EF1A2; soybean	U6-9-1	Particle bombardment	–	59–76%	Li et al., 2015
*Brassica oleracea*	BolC.GA4; ortholog of Arabidopsis GA4a	CsVMW; humans	At-U6-26	Agro-transformation of cotyledonary petioles	Golden gate	10%	Lawrenson et al., 2015
*Lotus japonicus*	SNF (symbiotic nitrogen fixation) related genes	2 × 35S	LjU6-1	*Agrobacterium* mediated stable or hairy root transformation	–	35%; biallelic	Wang et al., 2016
*Camelina sativa*	FAD2 gene; key enzyme for synthesis of polyunsaturated fatty acids	CaMV35SP	U9P	*Agrobacterium* floral dip transformation	Golden gate	60%	Jiang et al., 2016

## CRISPR/Cas9 System

**FIGURE 3:**
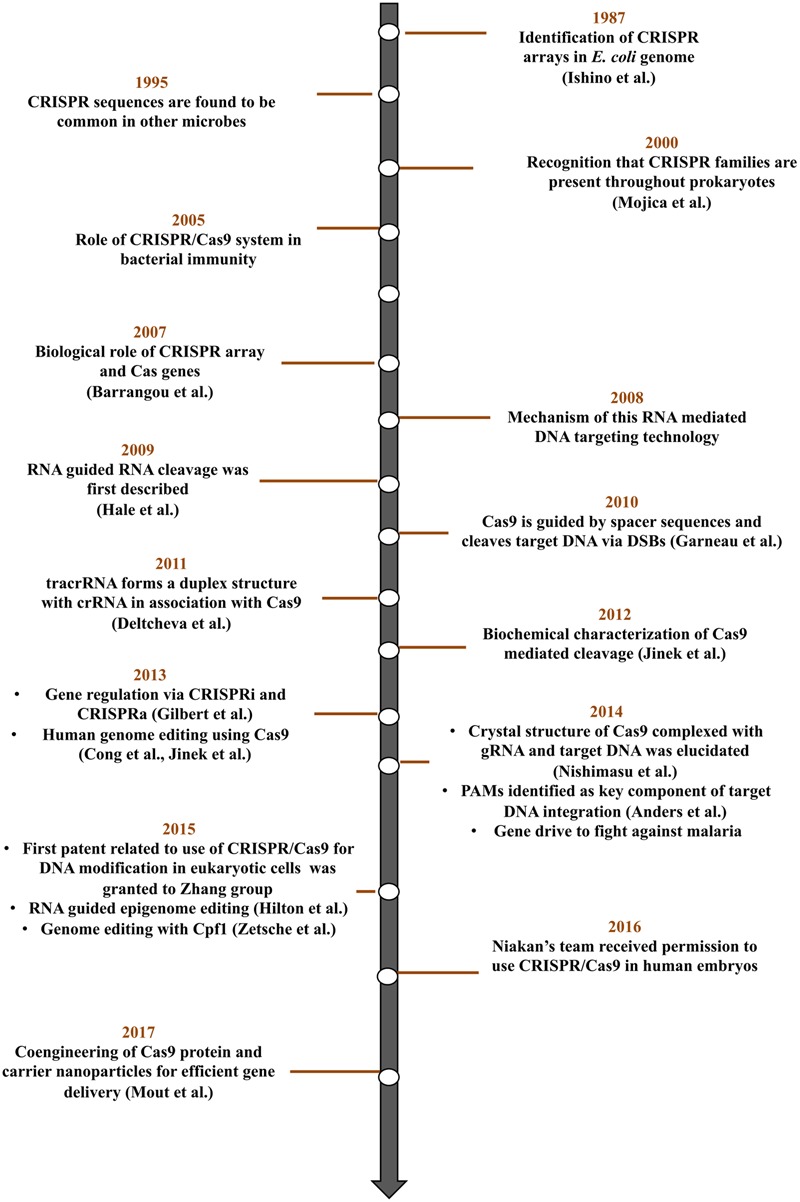
Key discoveries and advances in CRISPR/Cas9 technology.

CRISPR progress in today’s world as genome editing tool can be traced back to its origin in the late 1980s (Ishino et al., 1987) and a decade of extensive experimentation since 2005 (**Figure 3**). CRISPR/Cas9 microbial adaptive immune system and its progress till date is the outcome of the work of numerous researchers around the globe. A series of comprehensive reviews (Bortesi and Fischer, 2015; Amitai and Sorek, 2016; Puchta, 2016) gives the detailed information of each aspect of CRISPR/Cas technology.

Deciphering the role of CRISPR/Cas system in bacteria and archaea elucidated the power of this system as a genome-editing tool. A series of experiments involving bioinformatic tools unveiled various CRISPR/Cas components and their function in providing adaptive immunity to bacterial cells. A CRISPR locus consists of clusters of CRISPR-associated (Cas) genes and CRISPR arrays where all immunological memories are engraved (Barrangou et al., 2007). CRISPR array is a genomic locus having series of 21–40 bp repeat sequences (direct repeats) interspaced by 25–40 bp variable sequences (spacers) (Jansen et al., 2002; Tang et al., 2002). In 2005, three independent research groups (Bolotin et al., 2005; Mojica et al., 2005; Pourcel et al., 2005) hypothesized the role of spacer elements as traces of past invasions of foreign DNA that provide immunity against phage infection. They also noted that spacers share a common end sequence, now known as PAM. Barrangou et al. (2007) experimentally demonstrated the involvement of CRISPR arrays in resistance to bacteriophages in association with Cas genes. At every infection, new phage DNA gets incorporated into the CRISPR array building potential to fight the upcoming infection. Studies from Brouns et al. (2008) unveil the transcription of phage spacer sequences into small RNAs (crRNAs) that guide Cas proteins to the target DNA. The mechanism of interference based on RNA-mediated DNA targeting and the role of Cas9 in introducing DSBs at a precise position, three nucleotides upstream of PAM was also demonstrated (Marraffini and Sontheimer, 2008; Garneau et al., 2010). Further a trans-activating CRISPR RNA (tracrRNA) forms a duplex with crRNA and guides Cas9 to its target (Deltcheva et al., 2011). Fusion of the crRNA and tracrRNA to form a single, synthetic guide RNA further simplified the system (Jinek et al., 2012). Finally, Cong et al. (2013) reported the ability of Cas9 to facilitate homology directed repair with minimum mutagenic activity.

### Classification of CRISPR/Cas9 System

The first attempt to classify CRISPR/Cas system was done by Haft et al. (2005). He defined 45 CRISPR-associated (Cas) protein families that are categorized into core proteins (Cas1, Cas2, Cas3, Cas4, Cas5, Cas6), 8 CRISPR/Cas subtypes and RAMP (repair associated mysterious protein) module in prokaryotic genomes. Makarova et al. (2011) classified CRISPR/Cas systems into three types: type I, type II, and type III depending on the presence of signature Cas3, Cas9 and Cas10 proteins, respectively (**Table 2**). This system was divided into 10 subtypes depending on the presence of additional signature proteins. This three-type classification system is further modified into two class-five type classification systems depending on the type of signature proteins and CRISPR loci (Makarova et al., 2015). Major differences between CRISPR classes are based on the composition of crRNP complexes. Class 1 CRISPRs have multiple subunit effector complexes while class 2 CRISPRs concentrates most of their functions with single protein effectors. Class 1 CRISPR system, for example, have different nucleases for pre-crRNA processing, spacer sequence loading, and targeted cleavage processing. In class 2, a single protein performs all of these functions. Type IV and type V belongs to class I and class II systems respectively. Two subtypes of type V system and VI type is also recognized, elaborating the classification to two-class–six-type–19-subtype system (Shmakov et al., 2015; **Table 2**). Cas1 and Cas2 genes are ubiquitous in all CRISPR/Cas types (Makarova et al., 2011).

**Table 2:** Classification of CRISPR/Cas9 system.

Class	Type	Subtypes	Organism harboring respective types	Signature Cas proteins	Other core proteins
Class 1^∗^	I	I-A	*Archaeoglobus fulgidus*	Cas3, Cas8	Cas1, Cas2, Cas5, Cas6, Cas7
		I-B	*Clostridium kluyveri*	Cas3, Cas8	Cas1, Cas2, Cas5, Cas6, Cas7
		I-C	*Bacillus halodurans*	Cas3, Cas8	Cas1, Cas2, Cas5, Cas7
		I-D	*Cyanothece* sp.	Cas3, Cas10	Cas1, Cas2, Cas5, Cas6, Cas7
		I-E	*Escherichia coli*	Cas3, Cas8	Cas1, Cas2, Cas5, Cas7
		I-F	*Yersinia pseudotuberculosis*	Cas3, Cas8	Cas1, Cas2, Cas5, Cas6, Cas7
		I-U	*Geobacter sulfurreducens*	Cas3, Cas8	Cas1, Cas2, Cas5, Cas6, Cas7
	III	III-A	*Staphylococcus epidermidis*	Cas10	Cas1, Cas2, Cas5, Cas6, Cas7
		III-B	*Pyrococcus furiosus*	Cas10	Cas1, Cas2, Cas5, Cas6, Cas7
		III-C	*Methanothermobacter thermaautotrophicus*	Cas10	Cas5, Cas7
		III-D	*Roseiflexus* sp.	Cas10	Cas5, Cas7
	IV	IV	*Acidithiobacillus ferrooxidans*	Csf1	Cas5, Cas7
Class 2^∗^	II	II-A	*Streptococcus thermophilus*	Cas9	Cas1, Cas2
		II-B	*Legionella pneumophila*	Cas9	Cas1, Cas2
		II-C	*Neisseria lactamica*	Cas9	Cas1, Cas2
	V	V	*Francisella cf. novicida*	Cpf1	Cas1, Cas2
	VI	VI	*Leptotrichia shahii*	C2c2	Cas1, Cas2

*^∗^Makarova et al., 2011, 2015.*

CRISPR-Cpf1 (Class II, Type V CRISPR from *Prevoltella* and *Francisella1*) is an advanced tool that uses a single Cpf1 protein for crRNA processing, target site recognition, and DNA cleavage. Cpf1 is functionally conserved to Cas9 protein but differs substantially in many aspects. The differences are as follows: it is a ribonuclease that processes precursor crRNA; it recognizes a thymine rich (like 5′-TTTN-3′) PAM sites (Zetsche et al., 2015a). PAM sequence is located upstream of the protospacer sequence and tracrRNA is not required for guiding Cas9 to the target site. The most important characteristic of Cpf1 is the generation of 4 bp overhangs in contract to blunt ends produced by Cas9 (Zetsche et al., 2015a). These sticky ends would provide more efficient genomic insertions due to sequence complementarity into a genome. Among several proteins in the Cpf1 family, LbCpf1 from *Lachnospiraceae bacterium* ND 2006 and AsCpf1 from *Acidaminococcus* sp. BV3L6 act more effectively in human cells compared with other orthologs (Kim et al., 2016). Class 2 type VI is characterized by an effector protein C2c2 (Class 2, candidate 2). C2c2 contains two nucleotide binding (HEPN) conserved domains, which lacks homology to any known DNA nuclease (Abudayyeh et al., 2016). HEPN domains function as RNases, hence it is visualized as a new RNA targeting tool guided by a single crRNA which can be engineered to cleave ssRNA carrying complementary protospacers. Hence, C2c2 does not target DNA (Abudayyeh et al., 2016). C2c2 is similar to type III-A and III-B systems in having HEPN domains that are biochemically characterized as ssRNA specific endoribonucleases but there is a significant line of difference between these two types. Cas10- Csm in type IIIA and Csx in type III B have less target specificity and have to dimerize to form active sites. C2c2, in contrast, contains two HEPN domains and function as monomeric endoribonuclease (Abudayyeh et al., 2016). dCas9 analogs of C2c2, dC2c2 can be produced by alanine substitution of any of the four predicted HEPN domain. Further examination is required to clarify the mechanism of the C2c2 system and the class of pathogens against which it can protect bacteria. Currently, type VI system is found in *Carnobacterium gallinarum, Leptotrichia buccalis, L. shahii, L. wadei, Listeria newyorkensis, L. seeligeri, L. weihenstephanensis, Paludibacter propionicigenes*, and *Rhodobacter capsulatus* (Choi and Lee, 2016).

### CRISPR/Cas9 Mechanism

**FIGURE 4:**
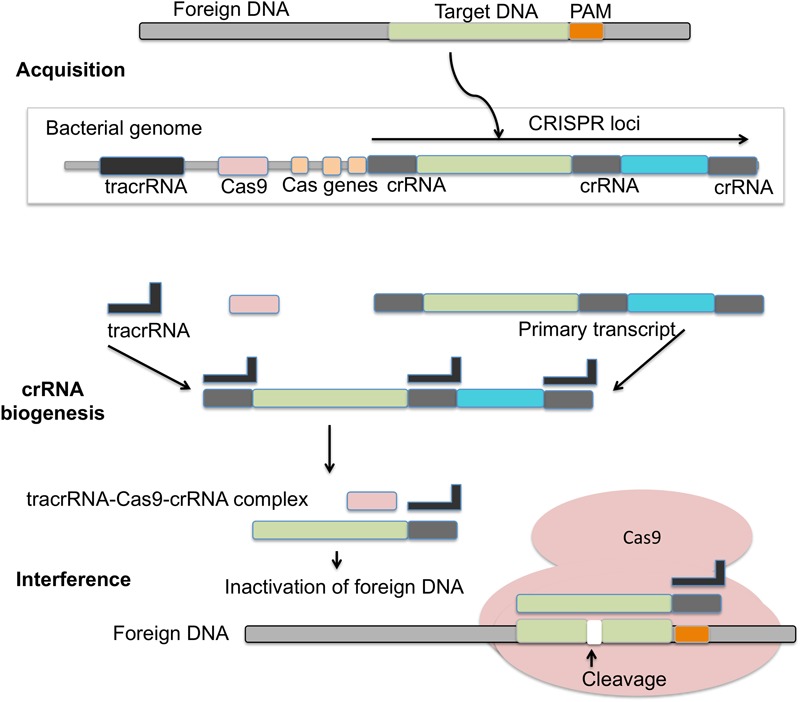
Mechanism of CRISPR/Cas9 action: in the acquisition phase foreign DNA gets incorporated into the CRISPR loci of bacterial genome. CRISPR loci is then transcribed into primary transcript and processed into crRNA with the help of tracrRNA during crRNA biogenesis. During interference, Cas9 endonuclease complexed with a crRNA and cleaves foreign DNA near PAM region.

The adaptive immunity of CRISPR/Cas9 system consists of three phases: adaptation, expression, and interference (**Figure 4**). Adaptation involves the invading DNA from virus or plasmids that are cleaved into small fragments and incorporated into CRISPR locus. CRISPR loci are transcribed and processed to generate small RNA (crRNA), which guide the effector endonucleases to target the viral material by base complementarity (Barrangou et al., 2007; Yosef et al., 2012). DNA interference in Type II CRISPR/Cas system requires a single Cas9 protein (Hale et al., 2009; Zetsche et al., 2015b). Cas9 is a huge protein possessing multiple domains (RuvC domain at the amino terminus and the HNH nuclease domain positioned in middle) and two small RNAs namely crRNA and tracrRNA. Cas9 assists adaptation, participates in pre-crRNA processing to crRNA and introduce targeted DSBs guided by tracrRNA and double stranded RNA specific RNase III (Jackson et al., 2014; Mulepati et al., 2014). As compared to type II CRISPR, the unique features of type III CRISPR are the cleavage of both DNA and RNA, and its association with the cleavage protein Cas10. The cleavage is a transcription-dependent DNA sequence modification that also contains a transcriptionally active promoter (Samai et al., 2015). Cas10 system enables bacteria to acquire viral spacer elements enabling a type of resistance against foreign DNA under special conditions. This resistance to foreign/viral DNA prevents activation of the lytic pathway, which is detrimental to the host cell. These sequences could also alter the physical characteristics of the cell, potentially providing a survival advantage for the host cell (Samai et al., 2015).

Multiplex genome engineering using multiple guide RNAs to target various genomic sites simultaneously was also demonstrated. CRISPR was first applied in plants in August 2013 (Feng et al., 2013; Li et al., 2013; Xie and Yang, 2013). Feng et al. (2013) targeted various endogenous genes and transgenes by protoplast transfection, agroinfiltration and generated stable transgenic plants by both NHEJ and HR mechanisms. Various genes leading to phenotypic variations were targeted like *Brassinosteroid Insensitive 1 (BRI1), Jasmonate-Zim-Domain Protein 1 (JAZ1)* and *Gibberellic acid insensitive (GAI)* in *Arabidopsis* and *Rice Outermost Cell-specific gene5 (ROC5), Stromal Processing Peptidase (SPP)*, and *Young Seedling Albino (YSA)* in rice and obtained positive results. Similarly, Xie and Yang (2013) introduced three guide RNAs at distinct rice genomic loci and analyzed the mutation efficiency of 3–8%. Off target mutations were also noticed but with minimum genome editing efficiency than the matched site.

Studies on maize (Liang et al., 2014), wheat (Wang et al., 2014), and sorghum (Jiang et al., 2013) provided an excellent foundation for the use of CRISPR in gene editing. These investigations postulated the first comprehensive data on parameters such as mutation efficiency, cleavage specificity, large chromosomal deletions and resolution of locus structure. Jiang et al. (2013) also demonstrated the expression of gRNAs under the control of multiple promoters. Fauser et al. (2014) emphasized the use of both CRISPR/Cas based nucleases and nickases with their studies conducted on *Arabidopsis thaliana*. Nucleases are efficient tools for NHEJ mediated mutagenesis and the combined action of two nickases can enhance recombination between tandemly arranged direct repeats, gene conversion guided by inverted repeats and can regulate mechanisms involving HR (Fauser et al., 2014). Single chimeric gRNA are found to be more efficient than individual crRNA and tracrRNA components (Miao et al., 2013; Zhou et al., 2014). Interestingly, four independent groups (Shan et al., 2013; Brooks et al., 2014; Zhang et al., 2014; Zhou et al., 2014) have demonstrated the introduction of biallelic or homozygous mutations in T1 generation of rice and tomato indicating the high efficiency of this system. The genetic changes are segregated normally in subsequent generations without further modifications (Zhou et al., 2014). Some examples of the CRISPR/Cas9 applications in plants are cited in **Table 1**.

CRISPR/Cas9 system is continuously being upgraded for better efficiency and specificity of gene targeting. The need for repurposing CRISPR/Cas9 system to alter eukaryotic genome has necessitated the addition of nuclear localization signals at one or both ends of the protein. The introduction of orthogonal CRISPR/Cas9 systems has broadened the application of this technology manifold. These orthologs include RNA guided endonucleases from *Streptococcus thermophilus* (St), *Neisseria meningitidis* (Nm), *Campylobacter jejuni* (Cj), and *Staphytococcus aureus* (Sa). Each orthogonal Cas9 system has unique specifications including variations in Cas9 proteins, PAM sites and gRNA scaffolds for target recognition (**Table 3**). Hou et al. (2013) demonstrated efficient targeting of endogenous genes in human pluripotent stem cells via NmCas9. They are the pioneers in the development of NmCas9 that uses 24-nucleotide (nt) protospacer to target DNA over 20 nt protospacer requirements of SpCas9 and StCas9. Extended PAM sequence (5′-NNNNGATT-3′) as compared to NGG sequence may further enhance the specificity.

**Table 3:** Cas9 variants with their origin and specifications.

Cas9 nuclease variants	Origin	PAM sites (5′ to 3′)	Specifications	Functions	Reference
Native Cas9 (SpCas9)	*Streptococcus pyrogenes*	NGG	100 nt long gRNA	Introduce double stranded breaks; create blunt ends	Mojica et al., 2005
Cas9 nickase (Cas9n)	Engineered from *S. pyrogenes*	NGG	Mutation in native Cas9 (RuvC or HNH D10, aspartate to alanine substitution)	Generate single stranded break; efficient HDR repair mechanism	Cong et al., 2013; Fauser et al., 2014
dCas9	Engineered from *S. pyrogenes*	NGG	Mutated Cas9	RNA guided transcription regulation (CRISPRi, CRISPRa); delivers GFP enabling visualization of genetic element dynamics	Hilton et al., 2015
Dimeric RNA-guided FokI nucleases (RFNs)	Engineered from *S. pyrogenes*	NGG	Fusion of dCas9 protein and FokI nuclease domain	High genome editing frequency and reduced off-target mutations	Tsai et al., 2014; Bortesi and Fischer, 2015
NmCas9	*Neisseria meningitidis*	NNNNGATT	Longer crRNA component (24 nt)	Reduced off-target effects	Hou et al., 2013
StCas9	*Streptococcus thermophilus*	NNAGAAW	On target cleavage activities	Reduced off-target effects	Horvath et al., 2008;
SaCas9	*Staphylococcus aureus*	NNGRRT or NNGRR(N)	On target cleavage activities	Reduced off-target effects	Esvelt et al., 2013
Cas9-DD (Destabilized Cas9)	*Engineered from S. pyrogenes*	NGG	Conjugation of destabilized domain to Cas9	Temporal, spatial and locus-specific control of gene expression; Increased NHEJ- mediated gene insertion efficiency	Geisinger et al., 2016; Senturk et al., 2015
Cpf1	*Prevoltella* and *Francisella1*	NTT	Contain a RuvC-like endonuclease domain, lack HNH endonuclease domain; 42 nt long gRNA	Require one RNA (crRNA); Produce staggered cut ends; easier to deliver in low capacity vectors ex. AAV	Zetsche et al., 2015a

## CRISPR Specifications in Plants

Efficient CRISPR/Cas9 genomes editing in plants require suitable vector system (codon optimized Cas9 gene and promoters for Cas9 and sgRNA), efficient target sites and transformation method used in appropriate plant species. CRISPR editing requires the delivery and expression of single guide RNA (sgRNA) and cas9 protein in the target cell. Specific expression vectors are designed to achieve this goal. sgRNA is generally regulated by tissue specific RNA polymerase III promoters such as AtU6, TaU6 etc. that drives the expression of small RNAs in their respective species. Similarly, Cas9 is placed downstream of RNA polymerase II promoters like ubiquitin promoters that guide the expression of longer RNAs. Cas9 is generally tagged with nuclear localization sequence (NLS) to target nuclear DNA. The choice of the vectors largely depends upon the type of the expression system to be worked on, type of restriction sites present to insert sgRNA and the type of Cas9 system. Both sgRNA and Cas9 can be co-expressed in a single plasmid ex. pFGC-pcoCas9, pRGEB32, pHSE401. Different types of plasmids can be studied from https://www.addgene.org/crispr/plant/. The use of these plasmids is limited depending upon the type of Cas9 (cut, nick, activate, interfere) present (**Table 4**).

**Table 4:** Different plasmids with specific Cas9 activity.

Crop	Plasmid	Gene/insert name	Promoter		Selectable Marker	Cas9 type	Significance	Reference
			sgRNA expression	Cas9 expression				
*Oryza sativa*	pRGEB32	PTG1/Cas9	U3 snoRNA	Rice ubiquitin	Hygromycin	Cut	Enhanced multiplex editing capability via endogenous tRNA processing system	Xie et al., 2015
*Zea mays*	pHSE401	gRNA scaffold	AtU6-26	35S	Hygromycin	Cut	Improved designing of CRISPR/Cas9 binary vector. Easy method for one or more gRNA assembly in expression cassette, high efficiency mutant generation	Xing et al., 2014
*Arabidopsis thaliana*	pKI 1.1R	Human codon optimized spCas9	U6-26p	CaMV35S, WOX2, RPS5A	Hygromycin	Cut	pKIR vector harboring *RPS5A* maintains high constitutive expression and heritable muatations at all developmental stages	Tsutsui and Higashiyama, 2017
*Arabidopsis, Nicotiana benthamiana, Oryza sativa*	pYPQ159	hSpCas9D10A (human codon optimized)	AtU6-26	2× 35S	Spectinomycin	Nick	This toolbox provides reagents to efficiently assemble DNA constructs for monocots and dicots using Golden Gate cloning	Lowder et al., 2015
*Zea mays, Arabidopsis*	pBUN501	zCas9D10A	AtU6-26	Ubi1	Bar	Nick	Multiplex genome editing	Xing et al., 2014
*Zea mays, Arabidopsis*	pHSN6A01	dCas9-VP64, gRNA scaffold	AtU6-26p	2× 35Sp	Hygromycin	Activate	Multiplex genome editing	Xing et al., 2014
*Zea mays, Arabidopsis*	pBUN6A11	dCas9-VP64, gRNA scaffold	OsU3p	Ubi1p	Bar	Activate	Multiplex genome editing	Xing et al., 2014
*Arabidopsis, Nicotiana benthamiana*	pEGB 35S:dCas9:Tnos (GB1191)	dCas9	AtU6-26	35S, Tnos	Kanamycin	Interfere	GoldenBraid 2.0 validation- foster DNA exchange for synthetic biology; protein-protein interaction tool, gene silencing tool	Perdigones et al., 2013
*Nicotiana benthamiana*	pdCas9 (GB1079)	Mutated Cas9 (D10A, H840A) and inactivated catalytic domains (human codon optimized)	U6-26	pNOS	Ampicillin	Interfere	GoldenBraid gRNA-Cas9 toolbox validated; multiple TDNA cloning, gRNA multiplexing; effective transcriptional activation and repression	Vazquez-Vilar et al., 2016

Independent sgRNA plasmids are also designed where Cas9 is not co-expressed but can be paired along enabling usage of the wide variety of Cas9 types. pICSL01009::AtU6p and pICH86966::AtU6p::sgRNA_PDS which encodes an *Arabidopsis* U6 promoter and expresses sgRNA targeting PDS in *Nicotiana benthamiana.* The choice of the optimal promoters to drive the expression of sgRNA or Cas9 and codon optimized version of Cas9 is important for efficient genome editing. Most of the work in eukaryotic cells is done using codon optimized versions of *SpCas9.* Results have been obtained using human codon optimized (Li et al., 2013; Miao et al., 2013) or plant codon optimized versions of Cas9 (Feng et al., 2013; Nekrasov et al., 2013; Xie and Yang, 2013). The mutations induced can be heterozygous, biallelic (two distinct allelic mutations), homozygous or rarely chimeric. A number of reports confirmed the stable inheritance of CRISPR/Cas9 induced mutations in model and crop plants. Efficient CRISPR/Cas9 genome editing and inheritance of modified genes in the T3 and T2 generations was reported for the first time in *Arabidopsis* (Jiang et al., 2014). A change in non-functional GFP gene was observed in T1 generation. All *GFP*-positive transgenic plants were identified with mutagenized *GFP* genes. Out of 42 transgenics developed, 50% have inherited a single T-DNA insert.

**FIGURE 5:**
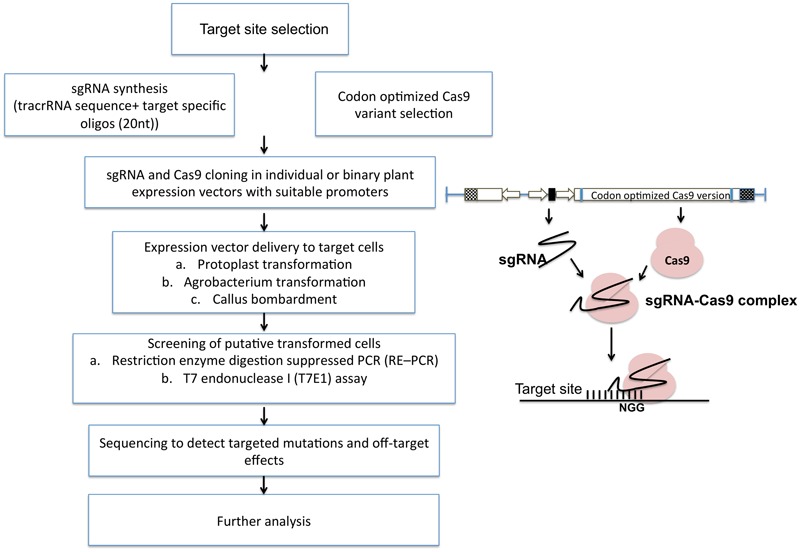
Simplified flow chart representing CRISPR/Cas9 mediated plant genome editing. After the selection of the target site, sgRNAs are designed using various bioinformatic softwares and packed into specific vectors along with codon optimized Cas9. After delivery into plant cells, putative transformants can be screened by multiple assays and used for further analysis.

The general methodology for implementing targeted mutagenesis using CRISPR/Cas9 technology is outlined in **Figure 5**. It starts with the selection of specific target site having a short PAM sequence at 3′ end. Target site should be selected considering minimum or no off-target effects (preventing cuts at unintended sites in the genome). Many bioinformatics tools help in designing sgRNA with high specificity and detection of off-targets such as COSMID (CRISPR Off-target Sites with Mismatches, Insertions, and Deletions). Off-targets are more prevalent in bacterial and cultured mammalian cells than in plant cells. Many studies have shown the potential off-targets of cas9 such as, in soybean, the off-target frequency was found to be 13% (Jacobs et al., 2015). No detectable off-targets are found in *A. thaliana*, wheat, rice and sweet orange. Cas9 nickase has also emerged as an alternative to reduce off target effects. Nickase is guided by the sgRNA at two adjacent positions at the target site producing a single stranded break on each of the two DNA strands.

CRISPR-PLANT is a newly designed web portal supported by PennState and Arizona Genomics Institute (AGI) established to help researchers to use the CRISPR-Cas9 system for genome editing. It estimates the highly specific sgRNA by avoiding off-target sequences (Xie et al., 2014). After the target site confirmation, target specific oligonucleotides (20 nt) are designed which further fuses with tracrRNA sequence to form sgRNA. sgRNA is further placed in a vector either along with Cas9 sequence (a binary vector) or individually under a suitable promoter for an optimal expression. The constructs are then transformed using a suitable method. The delivery systems vary based on plant species, research purpose, and requirements. F gRNA-Cas9 mediated editing can be detected by a restriction enzyme digestion suppressed PCR (RE-PCR) method, which investigates the NHEJ-introduced mutations (Xie and Yang, 2013). RE-qPCR can also be performed for more accurate estimation of genome-editing efficiency. Finally, whole genome sequencing is done to reduce off-target modifications.

## Targeted Genome Modification in Crop Plants

Over the years the biotic (bacteria, fungi, insects, and viruses) and abiotic (salinity, drought, flooding, heavy metal toxicity, high temperature) stresses have adversely affected crop plantation. One of the current researches in plant biology focuses on generating crops to tolerate harsh agro climatic conditions and to meet the needs of the ever-growing human race.

### Genome Modification for Nutrition Improvement

CRISPR/Cas9 system can generate stable and heritable mutations without affecting the existing valuable traits. This results in the development of homozygous modified transgene free plants in only one generation and it’s stable transmission to successive generations (Feng et al., 2014; Pan et al., 2016). Cas9 continued to be a better tool with relatively high cleavage efficiency when compared to TALENs and ZFNs (Gaj et al., 2013; Johnson et al., 2015). Researches done on various crops since the advent of CRISPR technology in the plant world is highlighted in **Table 1**. Classic works are being done for producing acrylamide free potatoes (Halterman et al., 2015), non-browning apples, mushrooms and potatoes by mutating Polyphenol oxidase (PPO) genes (Halterman et al., 2015; Nishitani et al., 2016; Waltz, 2016) and low phytic acid in maize (Liang et al., 2014).

Wang et al. (2014) pioneered the work of targeted genome editing in sweet orange using Cas9/sgRNA. Genetic improvement of citrus is limited due to its slow growth, pollen incompatibility, polyembryony, and parthenocarpy. Xcc (*Xanthomonas citri* subsp. *citri*)-facilitated agroinfiltration was employed to deliver Cas9 and CsPDS gene specific sgRNA into sweet orange. DNA sequencing confirmed the mutated CsPDS gene at the target site with a mutation rate of 3.2 to 3.9%. No off-target mutagenesis was reported. Lawrenson et al. (2015) targeted multicopy genes in *Hordeum vulgare* investigating the use and target specificity requirements of Cas9 editing. HvPM19 gene encoding an ABA-inducible plasma membrane protein was targeted to study the characteristics of dormancy. T_0_ were phenotypically identified with expected dwarf phenotype associated with a knockout of the target gene. Liang et al. (2014) discussed the presence of anti nutritional compound Phytic acid (PA), inositol 1,2,3,4,5,6-hexakisphosphate in maize. PA is poorly digested in humans and posses a threat to the environment, thus, PA content of maize seeds was reduced by designing two gRNAs targeting the ZmIPK (Inositol Phosphate Kinase) gene that catalyzes a key step in PA biosynthetic pathway.

### Biotic and Abiotic Stress Resistance via CRISPR/Cas9

Multiple disease resistance plants have been obtained using CRISPR/Cas9 technology (**Table 5**). Some highlights involve the resistance against rice blast disease by targeting *OsERF922* gene in rice (Wang et al., 2016). Transgene free mutant lines from T1 and T2 generations were selected by segregation and further examined. Transgenic lines showed a significant reduction blast lesions formed due to pathogen infection. Wang et al. (2014) introduced mutations using site-specific endonucleases in homeoalleles encoding *Mildew-resistance locus (MLO)* proteins of hexaploid bread wheat. Peng et al. (2017) targeted citrus canker caused by *Xanthomonas citri* subsp. *Xcc* in *Citrus sinensis.* CRISPR/Cas9 targeted modification of the susceptibility gene *Lateral organ boundaries 1* (*CsLOB1*) promoter enhances disease resistance. Deletion of the entire EBE_PthA4_ sequence from both *CsLOB1* alleles conferred the highest level of resistance to Wanjincheng orange. All transformed plants were morphologically similar to wild type indicating that *CsLOB1* promoter modification does not disrupt plant development. 42% of the mutant plants harbored desired mutations and 23.5% of the mutants showed resistance to citrus canker. The stacking up of multiple nucleases as one transgene by CRISPR/Cas9 system also leads to the targeted cleavage of multiple infections by viruses (Iqbal et al., 2016).

**Table 5:** List of some crops that are made resistant to diseases via CRISPR/Cas9 system.

Crop	Disease/symptoms	Causal/target organism	Targeted gene	Significance	Reference
*Triticum aestivum*	Powdery mildew disease	*Blumeria graminis f.* sp. *Tritici*	*TaMLO-A1* (wheat mildew resistance locus1)	Simultaneous modification in three homoeoalleles, heritable broad spectrum resistance to powdery mildew	Wang et al., 2014
*Oryza sativa*	Bacterial blight of rice	*Xanthomonas oryzae*	*OsSWEET11, OsSWEET14* (rice bacterial blight susceptibility genes)	PEG stimulated Cas9/sgRNA gene uptake in rice protoplast (*Agrobacterium* independent method), Cas9/sgRNA mutations occur within plant cells, free of bacterial cell involvement	Jiang et al., 2013
	Rice blast disease	*Magnaporthe oryzae*	*OsERF922* (ethylene responsive factor transcription factor)	42% T_0_ mutant lines; 6 T_2_ homozygous mutants showed high blast resistance and have same agronomic traits	Wang et al., 2016
*Arabidopsis thaliana*	Turnip mosaic virus disease	*Potyvirus* (TuMV)	*elF(iso)4E (elF transcription factor)*	Mutants show no growth defects, morphologically similar to wild type	Pyott et al., 2016
*Gossypium hirsutum*	Cotton leaf curl disease	*Begomovirus*	*CLCuD IR* and *Rep regions*	Targeted cleavage of mixed infections by multiple viruses and associated DNA satellites, such as CLCuD-complex	Iqbal et al., 2016
*Cucumis sativus L.*	Ring spot disease, vein yellowing disease	*Cucumber vein yellowing virus (Ipomovirus), potyviruses Zucchini yellow mosaic virus and Papaya ring spot mosaic virus-W*	*elF4E* (eukaryotic translation initiation factor 4E)	eLF4E disruption generated virus resistant heterozygous non-transgenic mutants	Chandrasekaran et al., 2016
*Nicotiana benthamiana*	Leaf thickening, chlorosis, curling	*Bean yellow dwarf virus (BeYDV)*	*BeYDV* (short intergenic region, trans acting replication initiation protein)	87% reduction in targeted viral load. Study proved that IR targeting via sgRNA confer better resistance	Baltes et al., 2014
	Leaf curl disease	*Tomato yellow leaf curl virus, Beet curly top virus*	*TYLCV-IR* (intergenic regions), *RCA regions*	Mutants showed delayed and reduced viral DNA accumulation	Ali et al., 2015

### CRISPR System in Metabolic Engineering

Further applications of CRISPR/Cas9 include extensive research in the field of metabolic engineering where plant cells are targeted for production of specific metabolites. Alagoz et al. (2016) manipulated the biosynthesis of benzylisoquinoline alkaloids (BIAs) for next generation metabolic engineering in *Papaver somniferum* by knocking out 3′ OMT2 gene via NHEJ DNA repair CRISPR/Cas9 mechanism. 4′ OMT2 (4′-*O*-methyltransferase) is a regulatory gene involved in the biosynthesis of codeine, noscapine, papaverine, and morphine via different BIA pathways. Such strategies can be employed to convert valuable medicinal plants into biofactories for mass production of specific metabolites simply by introducing breaks in related gene sequencing. Li et al. (2017) targeted *diterpene synthase gene (SmCPS1)*, involved in tanshinone biosynthesis in *Salvia miltiorrhiza*, Chinese herb well-known for vasorelaxation and antiarrhythmic effects. *SmCPS1* is the entry enzyme that uses GGPP (geranylgeranyl diphosphate) as its substrate for generating tanshinones. GGPP also acts as a precursor for taxol biosynthesis, therefore; SmCPS1 knockout (post-GGPP synthesis step) blocks the metabolic flux through GGPP to tanshinone, switching GGPP to taxol synthesis. *Agrobacterium rhizogenes* mediated transformation using CRISPR/Cas9 generated three homozygous and eight chimeric mutants from 26 independent transgenic hairy root lines of *Salvia*. Metabolomics analysis revealed zero tanshinone accumulation in homozygous mutants and decreased percentage in chimeric mutants.

## Prospective Applications of CRISPR System

**FIGURE 6:**
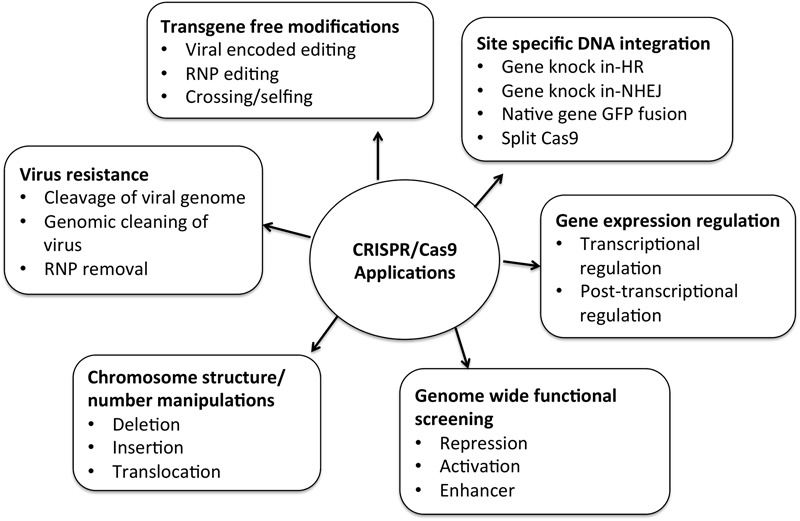
Various applications of CRISPR/Cas9 system many of which are yet to be tested in plants.

CRISPR/Cas9 technology is advancing at an unprecedented pace. Most of the research done so far include gene knockout or gene silencing mechanisms via NHEJ, which is not precise and most prevailing mechanism. Gene knock-in or gene replacement strategies that follow targeted mutagenesis via HDR evidenced promising results in mammalian and plant cells. Homology driven repair was a difficult task earlier in plants because of low efficiency and inefficient delivery of homologous donor sequences into transfected plant cells (Puchta and Fauser, 2014; Steinert et al., 2016). Multiple approaches are used for efficient homology directed repair mechanism and successful results have been reported (Collonnier et al., 2017; Humanes et al., 2017). Genomic studies in woody plants are challenging because of the long vegetative periods, low genetic transformation efficiency and limited mutants. Fan et al. (2015) reported the disruption of site-specific endogenous *phytoene desaturase gene (PtoPDS)* in *Populus tomentosa Carr. via*. Homoallelic and heteroallelic *pds* mutants were detected in first generation. CRISPR/Cas9 has also been applied to lower members of kingdom Plantae like algae, bryophytes, pteridophytes, etc. Liverworts emerge as model species for studying land plant evolution. Molecular genetics of *Marchantia polymorpha L.* is studied by the application of CRISPR/cas9 targeted mutagenesis (Sugano et al., 2014). Beyond genome editing, CRISPR/Cas9 technology is widely developing and used for various other purposes to understand functional genomics and molecular biology. The current focus is on loss-of-function and gain-of-function analysis of individual genes and identification of gene modules and genetic expression. **Figure 6** represents the expanding footprints of CRISPR/Cas9 system of which many are yet to be tested in plants.

CRISPR has replaced the RNA interference (RNAi) gene silencing technology for efficient and precise gene knock down. It has overcome various limitations of RNAi technology, such as incomplete loss-of-function analysis and extensive off-target activities. With the development of simultaneous expression of multiple guide RNAs (sgRNAs), CRISPR/Cas9 system allows, “multiplex genome editing.” Multiplex genome-editing acts as a powerful tool for reducing genetic redundancy in paralogous sequences by creating multiplex gene knockouts. It has also been used to create chromosomal deletions from multiple DNA base pairs in *Arabidopsis, Nicotiana benthamiana* etc.

### Gene Expression Regulation

Manipulating the genome of the target cells is another well-known CRISPR/Cas9 application. Repurposing CRISPR/Cas9 gene editing to gene expression regulation is known as CRISPR interference (CRISPRi). CRISPR interference involves either activation or repression of the gene expression (Bikard and Marraffini, 2013). The establishment of CRISPR/Cas9 as gene regulatory machinery came up majorly from experimental studies on intracellular pathogen *Francisella novicida*. FTN-0757 gene expresses the virulence factor that represses the production of a bacterial lipoprotein (BLP). FTN-0757 is further examined as a type II Cas9 protein that in association with tracrRNA inactivate BLP expression in *Francisella novicida*. tracrRNA has an imperfect complementarity to BLP messenger, which requires Cas9 and a small CRISPR-Cas-associated RNA (scaRNA) for BLP mRNA degradation (Bikard and Marraffini, 2013). A number of excellent reviews give the detailed information on principles of gene regulation by CRISPR/Cas system including (Larson et al., 2013; Qi et al., 2013; Xu et al., 2014; Lee et al., 2016).

Targeted regulation of gene expression provides interesting insights into the plant genome as well (Petolino and Davies, 2013). The ectopic gene expression regulation provides important information for gene functioning and can also be applied to develop regulatory circuits for synthetic biology applications (Puchta, 2016). Precise manipulation of the desired gene expression by repression or activation can elucidate the function of individual genes and their role in complex developmental processes (Dominguez et al., 2016). Gene expression regulation depends on the type of inducible or repressible promoters and the chemical or physical treatments for promoter activation and repression. Simultaneous multigene repression in plants was evaluated by Lowder et al. (2015). A synthetic repressor system (pCo-dCas9-3X- SRDX) was designed and tested on *Arabidopsis cleavage stimulating factor 64* (AtCSTF64) gene and on non-protein coding genes (redundant microRNAs- miR159A and miR159B). The multigene gRNA designed against these genes were constructed into a T-DNA cassette harboring pCo-dCas9-3X (SRDX) pUBQ10 control. The transcript levels were reduced approximately by 60% as compared to control among the three independent transgenic lines. Similarly, the transcript levels were reduced to 50% and more in transgenic lines expressing miR159A and miR159B targeting construct.

### Live Cell Imaging

**FIGURE 7:**
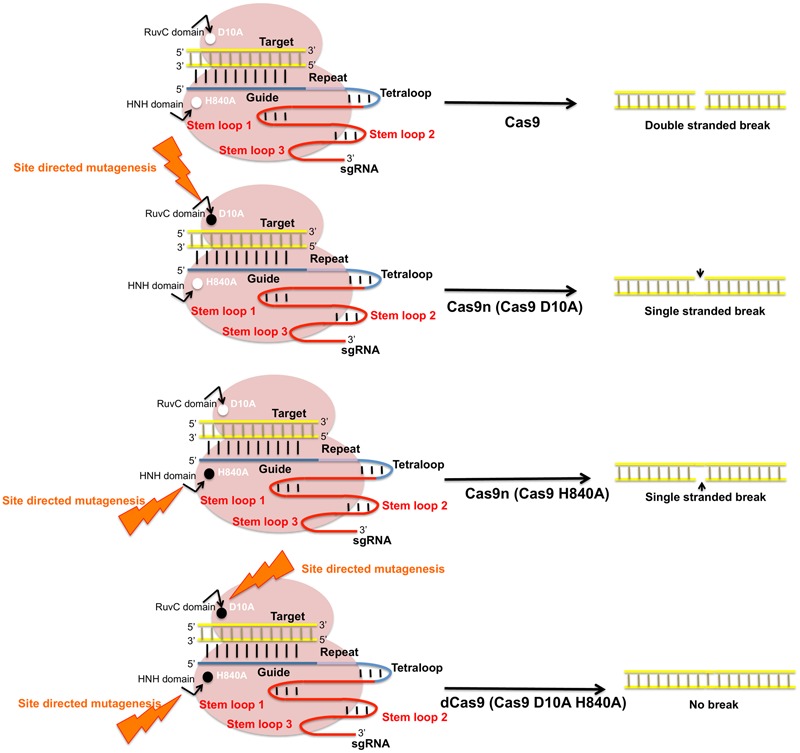
Schematic representation of Cas9 nuclease activity and its modifications. SpCas9 endonucleases create DSBs in target DNA through the activity of RuvC and HNH nuclease domains. SpCas9 nucleases can be converted into DNA nickase by substitution of its key amino acids D10A and H840A that produces single stranded breaks. Site directed mutagenesis in D10A produces Cas9n D10A and mutation in HNH domain produces Cas9n (H840A). Mutations in both catalytic residues modify Cas9 to an inactive dead Cas9 (dCas9).

Plant chromosomes are highly organized and compact structures. The spatiotemporal organization of plant genome determines the regulatory characteristics of various cell functions such as DNA replication and repair, transcription and cell death. Studies analyzing subcellular localization of genes and change in chromosomal structures provide insights into genome regulation and the systemic regulation of coding and non-coding genes during development. *In vivo* visualization of the defined DNA sequences is done prior by fluorescent *in situ* hybridization (FISH) but CRISPR imaging has overcome various issues related to FISH such as its inability to visualize dynamic processes and the requirement of fixed tissue samples. FISH also requires the cell fixation and DNA denaturation step which may alter the chromatin structure (Boettiger et al., 2016). CRISPR/Cas9 technology is customized with the introduction of Cas9 variant known as “dead Cas9” (dCas9). dCas9 is a catalytically inactive form of the nuclease (point mutation in either of the two catalytic domains, HNH and RuvC) that fuses with general transcription factors to its C-termini (**Figure 7**). dCas9 has the ability to bind to specific target DNA guided by sgRNA and allows direct imaging and manipulation of transcription without altering the DNA sequence (Dominguez et al., 2016). Puchta (2017) developed a CRISPR-dCas9 based cell imaging technique based on site directed mutagenesis of two Cas9 orthologs derived from *Streptococcus pyrogens* (Sp-dCas9) and *Staphylococcus aureus* (Sa-dCas9) followed by fusion of multiple copies of fluorescence proteins to the C-terminal end of each dCas9 variant. The use of dCas9 to inhibit gene expression is referred as CRISPR interference (CRISPRi). It is also used to deliver specific cargos and effector proteins to targeted genomic loci for transcriptional gene regulation. dCas9 has also being utilized to recruit transcriptional activators to the target promoter (Bikard et al., 2013). Gene activation and repression in plants is still advancing with positive results reported in *Nicotiana benthamiana* (Piatek et al., 2015) and *A. thaliana* (Lowder et al., 2015). This new Cas9 based system can further be employed to control the spatiotemporal patterns of gene expression in plants and modulating life cycles of various economically useful crops (Yang, 2015).

### Generation of Mutant Libraries

A genomic library is an indispensable tool to identify gene function by assessing the cellular phenotypes of loss of function mutants. Progression of genetic mutant libraries has simplified the genomic explication of gene function in multiple organisms (Schaeffer and Nakata, 2016). Genetic perturbations can be achieved via conventional approaches like altering the copy number of the gene, mRNA or protein; use of chemical mutagens; irradiation (Ahloowalia and Maluszynski, 2001); or random integration of foreign DNAs (Tadege et al., 2008). cDNA libraries for gain-of-function mutations and short interfering (si)RNA libraries for loss-of-function mutations are considered as high-throughput screening approaches but have various drawbacks like lack of control of over expression levels and obstinate downstream analysis due to mutation at multiple loci (Agrotis and Ketteler, 2015). Now CRISPR/Cas9 is repurposed to enable high throughput sequence screening. Functional screening is generally done in two formats- arrayed and pooled (Shalem et al., 2015). Various publications illustrate the role of CRISPR/Cas9 technology in screening. The arrayed format is a one gene per well-analyzing tool. Individual reagents are arranged in multiwell plates with a single reagent per well (Shalem et al., 2015). Since each reagent is prepared separately, this method is expensive and time-consuming but allows investigation of a wider range of cellular phenotypes. Pooled libraries are single preparations of many different plasmids. These screens are less expensive and labor intensive (Shalem et al., 2015).

### DNA Free Modifications of Plant Genome

Cas9 edited crops are assumed to cross many hurdles and issues to be classified as genetically modified crops. Generally, CRISPR/Cas9 DNA constructs are delivered into plant cells by *Agrobacterium*-mediated infiltration (Li et al., 2013), particle bombardment (Miao et al., 2013) and protoplast transfection (Shan et al., 2013). The *Agrobacterium*-mediated method is more popular because it has a propensity to insert single or a low copy number of transgenes and does not require an expensive particle gun apparatus (Char et al., 2016). However, the extra DNA delivered along the gRNA, Cas9 and selectable marker genes frequently integrate into the plant genome and may cause side effects like gene disruption, plant mosaicism and off target disruptions. Foreign DNA molecules can further integrate into the targeted DSB sites, lessens the efficiency of gene editing and gene insertion.

**FIGURE 8:**
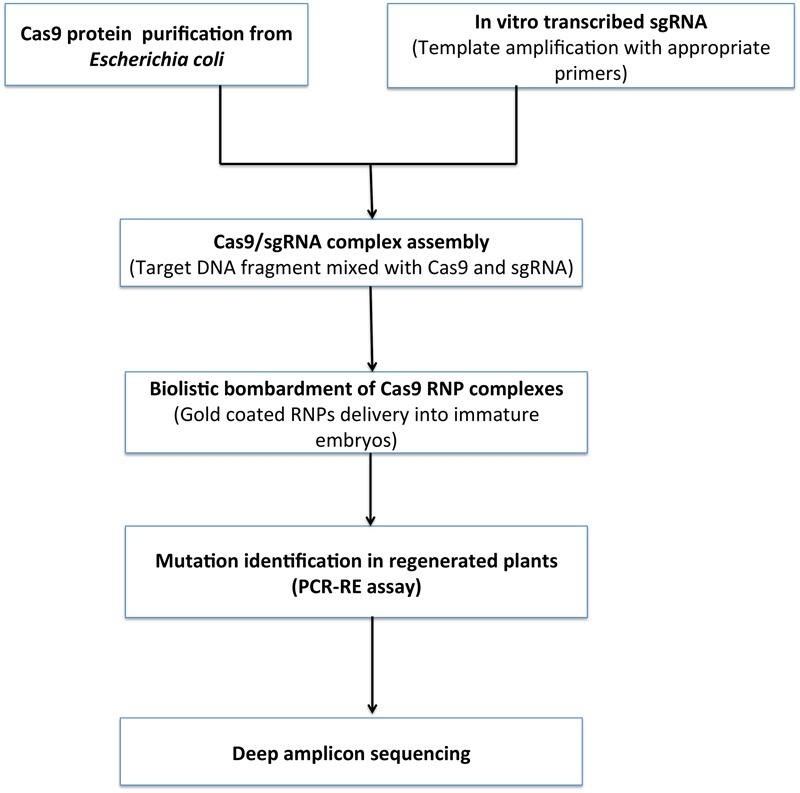
Proposed workflow for DNA free genome editing. Cas9 is expressed purified from E. coli. *In vitro* transcription of single guide RNA (sgRNA) and transcribed *in vitro* and RNP complex formation. RNPs and DNA precipitation onto 0.6 μm gold particles followed by Particle bombardment in targeted cells. Plants regeneration without any selective agent from bombarded cells and screened for mutations via PCR/restriction enzyme assay and deep sequencing.

To alleviate the disadvantages of plasmid based expression of Cas9/gRNA; efficient DNA-free genome editing is adopted which uses Cas9 ribonucleoproteins (RNPs). Cas9 RNPs are *in vitro* pre-integrated Cas9 nucleases and gRNA that are delivered into plant cells as RNA molecules (**Figure 8**). Cas9 RNPs are equally efficient to plasmid based expression systems for gene knockouts and gene editing. These ribonucleoproteins can be delivered in mammalian cells via lipid-mediated electroporation or transfection techniques (Liang et al., 2015). However, in plants the presence of cell wall hinders these techniques. Therefore, RNPs are delivered in isolated plant protoplasts and successful results have been obtained in a variety of plants such as tobacco, Arabidopsis, lettuce, rice, Petunia, and wheat (Woo et al., 2015; Subburaj et al., 2016; Zhang et al., 2017).

Similarly, Malnoy et al. (2016) have targeted the *MLO-7* gene in grapes for developing resistance against powdery mildew disease and *DIPM-1, DIPM-2*, and *DIPM-4* genes in apple for resistance against fire blight disease using CRISPR/Cas9 ribonucleoproteins. Commercially available recombinant Cas9 protein (160 kDa) was used and sgRNA was designed via CRISPR RGEN tools website for target specific sites having higher out of frame scores to achieve maximum knock out efficiency. Direct delivery of CRISPR RNPs in plant protoplast and efficient targeted mutagenesis with 0.1% and 0.5–0.69% indels (insertion or deletion) in targeted sites of *MLO-7* and *DIPM*-1, 2, and 4 was reported respectively. But plant regeneration from protoplast is challenging for most of the cereal crops, mainly monocots. Therefore, DNA-free efficient genome editing has been done in multiple crops like rice, maize, wheat using CRISPR/Cas9 ribonucleoprotein complexes via particle bombardment in embryo cells. Svitashev et al. (2016) are the pioneers to report biolistic delivery of Cas9-gRNA RNP into immature maize embryo. *Liguleless 1 (LIG)* gene, *Male fertility genes (MS26 and MS45)* and *Acetolactate synthase gene (ALS2)* was targeted and mutation frequencies of Cas9/gRNA plasmid based system and Cas9 RNPs were evaluated. Mutation frequencies of plasmid based Cas9 system- 0.004, 0.020, 0.004, and 0.002% respectively for *LIG, ALS2, MS2*6, and *MS45* was remarkably low when compared to RNP delivery where the frequencies were 0.57, 0.45, 0.21, and 0.69 respectively. Finally, efficient delivery and high cleavage activity of RNPs was demonstrated (Svitashev et al., 2016).

## CRISPR/Cas9 Opportunities and Concerns

Customizable sequence specific nucleases are a powerful tool for plant genome editing. Historically, mega nucleases, ZFNs, and TALENs have been SSNs of choice but the introduction of CRISPR/Cas9 system has revolutionized the genome editing technologies. The importance of this system lies in its relative ease of use, high precision, and low start-up cost. The most distinct feature of CRISPR technology, i.e., DNA cleavage recognition through Watson and Crick base pairing drastically simplifies the DNA targeting. The emergence of two RNA components (CRISPR RNA and trans activating CRISPR RNA) into sgRNA has further simplified the CRISPR/Cas system and enhanced reagent delivery (Jinek et al., 2012; Cong et al., 2013). CRISPR/Cas system allows simultaneous targeting of multiple genomic loci due to the simplified engineering of target specificity (Zhou et al., 2014). Moreover, CRISPR/Cas system can readily be engineered to Cas9 nickases, introducing single stranded breaks. Compared to zinc finger nickases and transcription activator-like effector nickases, Cas9 nickases have no residual nuclease activity and greatly alleviate the risk of off-target activity.

Advancements and characterization of new CRISPR effector proteins have broadened the range of biotechnological applications via CRISPR/Cas system. For example, dormancy in any cells such as cancer cells can be achieved using type VI C2c2 effector proteins. C2c2 can inhibit cell growth *in vivo* when primed with cognate RNA (Abudayyeh et al., 2016). The potential of an inactive programmable RNA- binding protein (dC2c2) can be used to track and visualize specific RNAs and to modulate the function of effector modules that can be used for the construction of synthetic regulatory circuits and large-scale screening (Abudayyeh et al., 2016). The continuous development and validation of new functional toolkits provide immense opportunities to activate an imprinted gene and gene expression. Lowder et al. (2015) have developed a multifaceted toolkit consisting of Golden Gate and Gateway compatible vectors. They demonstrated the less explored multiplexing by expressing three independent gRNAs simultaneously in tobacco, rice, and Arabidopsis and successfully triggered or suppressed the expression of protein coding and non-coding genes. Kleinstiver et al. (2015) proposed a solution to gRNA mismatch and off-target editing by featuring the interaction between four different domains of Cas9. These domains increase the binding energy of Cas9 to targeted sequences up to mismatches, thus weakening these interactions would provide better results to improve off-target interactions. Major limitations of the CRISPR/Cas9 system include inefficient HDR to NHEJ ratio and very few simultaneous changes per cell. The frequent occurrence of non-target effects further hampers the use of this technology. One of the major drawbacks of Cas9 editing is mismatched cleavage when the gRNA mismatches a few bases. Many reports indicated the infrequency with which CRISPR cuts the non-targeted sequences (Fu et al., 2013; Hsu et al., 2013).

## Visionary Notions of this Technology

Research investigation in the past quadrennial has transcend genome editing tools ranging from targeted gene modifications to designing eIF4E resistance alleles which is a key player in virus resistance (Bastet et al., 2017) to alter genes to create multiple attributes like tolerance to abiotic and biotic stress in plants viz. drought tolerance, virus and disease resistance, enhanced nutritional, high yield crop and enhance shelf-life of the plants. CRISPR-Cas9 technology witnesses the future of versatile genome editing with robust and efficacious consequences. The forte of gene editing in plants including crops has been radically changed by CRISPR-Cas9 technology. Exploring the fundamental biology of plant development and stress response will facilitate in designing elite and superior crops. The CRISPR-Cas9 holds a very promising future in making designer plants by taking only the gene of interest from a wild type species and the gene is then directly interpolated at a precise location, which in turn opens many avenues for plant breeders for making designer plants. Various approaches are going to design plants in such a manner, which could withstand with all possible harsh challenges. The newly emerged CRISPR/Cas9 RNP system evaded the need to relay on target cell potential for Cas9 translation and its plausible meeting with gRNA.

CRISPR/Cas9 sequence specific nuclease editing is an effective approach to combat rice blast disease (Wang et al., 2016). OsERF922 gene in rice was targeted and 21 CRISPR-ERF922 induced mutants were identified from 50 T_0_ transgenic plants (Wang et al., 2016). Furthermore, the high throughput can be obtained by coalescence of cytidine deaminase enzyme with Cas9, which permits high-efficiency emendation of target codons in rice (Li L. et al., 2016). dCas9 fusion with cytidine deaminase allows direct conversion of cytidine to uridine leading to a point mutation from C/G bp to T/A bp during replication in one of the daughter cells (Puchta, 2016). Researches in the advancement of legendary technology are deliberately going on but one stubborn and constantly following pitfall related to off-targets in plants, which could be executed by doing whole genome sequencing. Many companies are also engaged in using this technology for the production of elite food and feed crops. The products, which are obtained by editing through CRISPR-Cas9, have no exogenous DNA and furthermore editing can be done in such a way, which abides by all the rules and regulations that are complaisant to withstand against Genetically Modified issues and can get an easy approval by the Department of Agriculture (USDA). In conclusion, CRISPR-Cas9 technology boasts of a promising future in making the desired mutation in plants because it has transformed and metamorphosed our potential to modify and regulate prokaryotic and eukaryotic genomes. The prevalent use of this technology will surely expedite its pace.

## Author Contributions

LA has written the manuscript under the supervision and drafting of AN. The review was finally edited and submitted by AN.

## Conflict of Interest Statement

The authors declare that the research was conducted in the absence of any commercial or financial relationships that could be construed as a potential conflict of interest.
